# Tissue factor expression in human pterygium

**Published:** 2011-01-08

**Authors:** Ryo Ando, Satoru Kase, Tsutomu Ohashi, Zhenyu Dong, Junichi Fukuhara, Atsuhiro Kanda, Miyuki Murata, Kousuke Noda, Nobuyoshi Kitaichi, Susumu Ishida

**Affiliations:** 1 Laboratory of Ocular Cell Biology and Visual Science, Department of Ophthalmology, Hokkaido University Graduate School of Medicine, Sapporo, Japan; 2 Ohashi Eye Center, Sapporo, Japan; 3 Department of Ocular Inflammation and Immunology, Hokkaido University Graduate School of Medicine, Sapporo, Japan; 4 Department of Ophthalmology, Health Sciences University of Hokkaido, Sapporo, Japan

## Abstract

**Purpose:**

A pterygium shows tumor-like characteristics, such as proliferation, invasion, and epithelial–mesenchymal transition (EMT). Previous reports suggest that tissue factor (TF) expression is closely related to the EMT of tumor cells, and subsequent tumor development. In this study, we analyzed the expression and immunolocalization of TF in pterygial and normal conjunctival tissues of humans.

**Methods:**

Eight pterygia and three normal bulbar conjunctivas, surgically removed, were used in this study. Formalin-fixed, paraffin-embedded tissues were submitted for immunohistochemical analysis with anti-TF antibody. Double staining immunohistochemistry was performed to assess TF and alpha-smooth muscle actin (α-SMA) or epidermal growth factor receptor (EGFR) expression in the pterygia.

**Results:**

Immunoreactivity for TF was detected in all pterygial tissues examined. TF immunoreactivity was localized in the cytoplasm of basal, suprabasal, and superficial epithelial cells. The number of TF-immunopositive cells in pterygial epithelial cells was significantly higher than in normal conjunctival epithelial cells (p<0.001). TF immunoreactivity was detected in α-SMA-positive or -negative pterygial epithelial cells. EGFR immunoreactivity was detected in pterygial epithelium, which was colocalized with TF.

**Conclusions:**

These results suggest that TF plays a potential role in the pathogenesis and development of a pterygium, and that TF expression might be involved through EMT-dependent and -independent pathways.

## Introduction

A pterygium represents an epithelial and fibrovascular configuration on the ocular surface adjoining the conjunctiva. The pterygium invades the cornea forming a wing-like shape, causing visual loss. Pathologically, a pterygium is a proliferative, invasive, and highly vascularized tissue [1]. Furthermore, there are transformed cells in pterygial tissue, which is one of the characteristics of a tumor phenotype [2]. Kase et al. [3,4] demonstrated that proliferation activity is high in the pterygial epithelium compared to that in the normal conjunctiva.

The phenomenon of epithelial cells changing their phenotype to fibroblastic cells after morphogenic pressure from injured tissue is called epithelial–mesenchymal transition (EMT) [5,6]. To develop highly invasive characteristics, epithelial tumor cells change their morphology and function, whereby they transiently acquire markers of mesenchymal differentiation (e.g., alpha-smooth muscle actin (α-SMA)), and lose some of their epithelial features (e.g., E-cadherin) [7]. Moreover, blockade of E-cadherin in cultured cancer cells similarly leads to changes in cell shape reminiscent of EMT, and this transition gave rise to cells with a highly metastatic phenotype. It has been demonstrated that E-cadherin immunoreactivity is involved in α-SMA-positive pterygial epithelial cells [4,8], suggesting that EMT plays a key role in the pathogenesis of pterygium.

Tissue factor (TF) is a transmembrane protein that interacts with coagulation factor VIIa, whereby it initiates blood coagulation. This interaction also triggers intracellular signals, which are primarily mediated by G protein–coupled protease-activated receptors in concert with adhesion molecules and several other factors [9]. TF is regulated by oncogenic and differentiation pathways and it functions in tumor initiation, tumor growth, angiogenesis, and metastasis [9-11]. Indeed, it has been demonstrated that epithelial tumor cells, expressing high levels of TF regulated by the differentiation pathway, have mesenchymal characteristics [9]. These results suggest that TF expression is closely related to the EMT of tumor cells, and subsequent tumor development.

The aim of this study was to analyze the expression and immunolocalization of TF in pterygial and conjunctival tissues in humans.

## Methods

### Preparation of human tissues

Eight patients with primary nasal pterygia who underwent surgical excision were enrolled in this study. Normal bulbar conjunctival tissues were obtained from three patients during cataract surgery. The tissues were then fixed in 4% paraformaldehyde. After fixation, slides were washed in phosphate-buffered saline and processed for paraffin sectioning. Informed consent was obtained according to the Declaration of Helsinki. All human experiments conformed to the requirements of ethics committee in Hokkaido University Graduate School of Medicine.

### Immunohistochemistry

Dewaxed paraffin sections were immunostained using the alkaline phosphatase complex method. Formalin-fixed, paraffin-embedded serial tissue sections were cut at a 4 μm thickness and endogenous peroxidase activity was inhibited by immersing the slides in 3% hydrogen peroxide in methanol for 10 min. As a pretreatment, microwave-based antigen retrieval was performed in phosphate-buffered saline (PBS). Then, non-specific binding of the primary antibody was blocked by incubating the slides in blocking bovine serum for 30 min. The slides were serially incubated with anti-TF monoclonal antibody (1:50; American Diagnostic Inc., Stamford, CT) for 2 h at room temperature, followed by a biotin-conjugated goat anti-mouse IgG. Positive signals were visualized using diaminobendizine as a substrate. In double staining immunohistochemistry, the sections were incubated with the above-mentioned first antibody, followed by the rhodamine-conjugated secondary antibody for 30 min, and FITC-conjugated anti-α-SMA monoclonal antibody (1:50; Abcam, Tokyo, Japan) for 30 min at room temperature. After washing, sections were mounted with mounting media with 4’,6-diamino-2-phenylindole (DAPI; SlowFade^®^ Gold antifade reagent with DAPI; Invitrogen, Eugene, OR). Preretinal fibrovascular membranes of proliferative diabetic retinopathy served as positive controls for TF immunohistochemistry [12]. In microscopic observation, we counted the number of epithelial cells and TF-positive cells of pterygium or normal conjunctiva in three fields under high power field (objective lens 40×). Cells positively stained for anti-TF antibody were noted by their labeling index as a percentage (%) in each specimen, and the measurements were averaged. The results regarding TF in pterygial tissues are presented as the mean.

To investigate the hypothesized co-localization of TF and EGFR in pterygium tissues, double staining immunohistochemistry was performed using mouse monoclonal antibody against human TF (1:50; Abcam) and anti-rabbit epidermal growth factor receptor (EGFR) polyclonal antibody (1:100 dilution; Santa Cruz Biotech, Santa Cruz, CA) as the primary antibody. Binding of the primary antibody was localized with the Alexa Fluor® 488 goat anti-mouse antibody (1:100 dilution; Invitrogen, Carlsbad, CA) and Alexa Fluor® 546 goat anti-rabbit secondary antibody (1:200 dilution; Invitrogen) for 30 min, respectively. Finally, sections were mounted with mounting media with DAPI.

### Western blot analysis

A pterygium and a normal conjunctiva were surgically removed, and then were sonicated in lysis buffer (1× RIPA buffer; Cell Signaling Technology, Danvers, MA) with protease inhibitor (Roche, Basel, Switzerland) on ice, and centrifuged at 1,3850× g for 20 min at 4 °C. These are stored in −80 °C until assayed. These samples were electrophoretically separated on SDS–PAGE using a 4% stacking and 10% separating gels. Proteins in gels were electro-transferred (80 V, 90 min, 4 °C) to Hybond-P polyvinylidene difluoride transfer membranes (GE Healthcare, Buckinghamshire, UK). After transfer, the membranes were incubated for 1 h in a blocking solution which consisted of 1% skim milk powder in PBS containing 1% tween (PBST), washed briefly in PBST, then probed with anti-TF monoclonal antibody (1:500; above described) or anti-α-SMA polyclonal antibody (1:500; Abcam, Cambridge, UK) diluted in 5% BSA/TBST. Membranes were extensively washed in PBST for 30 min and incubated with a 1:1000 dilution of the appropriate horseradish peroxidase-conjugated donkey anti-mouse or anti-rabbit IgG at room temperature for 60 min. Then placed in chemiluminescent reagent (ECL plus, GE healthcare, Buckinghamshire, UK) and exposed to luminescent image analyzer (Fujifilm, Tokyo, Japan).

### Statistical analysis

Student’s t*-*test was used for statistical comparison of the number of TF-immunopositive epithelial cells between pterygium and normal control groups. Differences between the means were considered significant when the probability values were <0.05.

## Results

**Figure 1:**
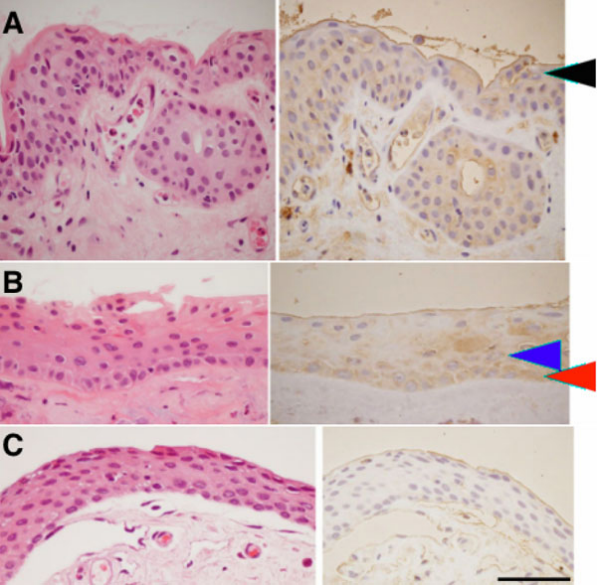
Immunohistochemistry for tissue factor (TF) in a human pterygium and normal conjunctiva. Left panels are H&E staining and right panels are TF immunoreactivity in two representative cases of a pterygium. TF is expressed in the cytoplasm of basal (**B**; red arrow head), suprabasal (**B**; blue arrow head), and superficial cells (**A**; black arrow head). In the normal conjunctiva, however, immunoreactivity for TF is not detected (**C**). The scale bar represents 50 μm.

Morphologically, pterygial epithelium consisted of multilayer nuclei showing squamous metaplasia (Figure 1A). Table 1 summarizes the immunohistochemical results of TF in pterygial epithelium. Immunoreactivity for TF was detected in all pterygial tissues examined. TF immunoreactivity was localized in the cytoplasm of basal, suprabasal, and superficial epithelial cells, and in subepithelial stroma along with epithelium (Figure 1A,B). In the normal conjunctival epithelium, however, immunoreactivity for TF was not detected (Figure 1C). Microvascular endothelial cells showed a weak immunoreaction for TF in both normal conjunctiva and pterygium. The number of TF-immunopositive cells was significantly higher in pterygial epithelial cells than in normal cells (p<0.001; Table 1).

**Table 1:** The number of tissue factor (TF)-immunopositive cells in pterygial epithelial and normal conjunctival cells.

**Pterygia**			**Normal conjunctivas**		
**Age**	**Gender**	**TF**	**Age**	**Gender**	**TF**
72	M	12.7%	54	M	0%
83	M	54.8%	80	M	0%
75	F	47.7%	82	M	0%
72	M	69.8%			
71	M	69.1%			
68	M	84.3%			
77	M	45.0%			
69	M	54.9%			
	Mean	54.8%		Mean	0%

In the Table, M indicates male and F indicates female.

**Figure 2:**
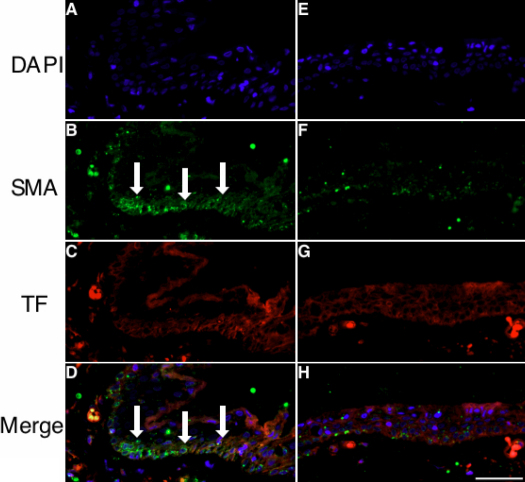
Double staining immunohistochemistry was performed for TF (red) and α-SMA (green) in pterygial tissue. **A**-**D**: α-SMA immunoreactivity is colocalized with TF-positive areas in pterygial epithelial cells (**D**, arrows). **E**-**H**: TF immunoreactivity is detected in the other part of epithelial cells negative for α-SMA. The scale bar represents 50 μm.

Double staining immunohistochemistry involving pterygial tissues was performed for TF and α-SMA expression. α-SMA was expressed in several epithelial cells (Figure 2B,F), where TF immunoreactivity was colocalized (Figure 2C,G). TF immunoreactivity was also detected in α-SMA-negative epithelial cells (Figure 2E-H).

**Figure 3:**
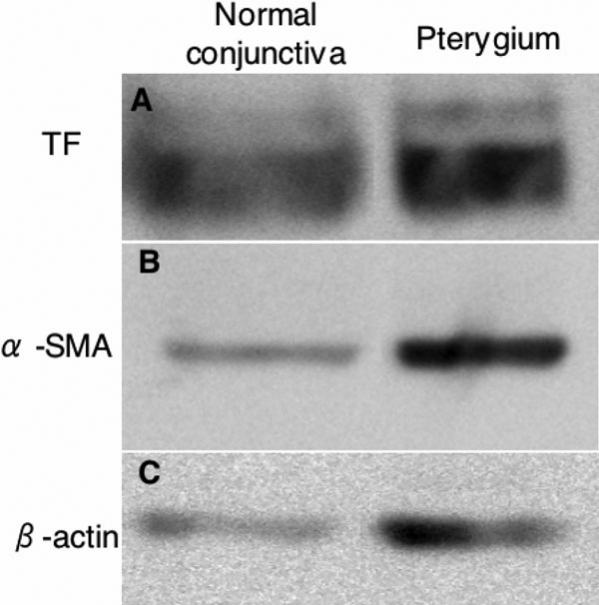
Western blot analysis using anti-TF and α-SMA antibodies. TF (**A**) and α-SMA (**B**) protein expression is clearly detected in both pterygium and normal conjunctival tissue.

To check the expression of TF and α-SMA by other methods in human pterygium and normal conjunctiva, western blot analysis was performed using anti-TF and α-SMA antibodies. TF and α-SMA protein expression was clearly detected in both total proteins extracted from pterygium and normal conjunctival tissues (Figure 3).

**Figure 4:**
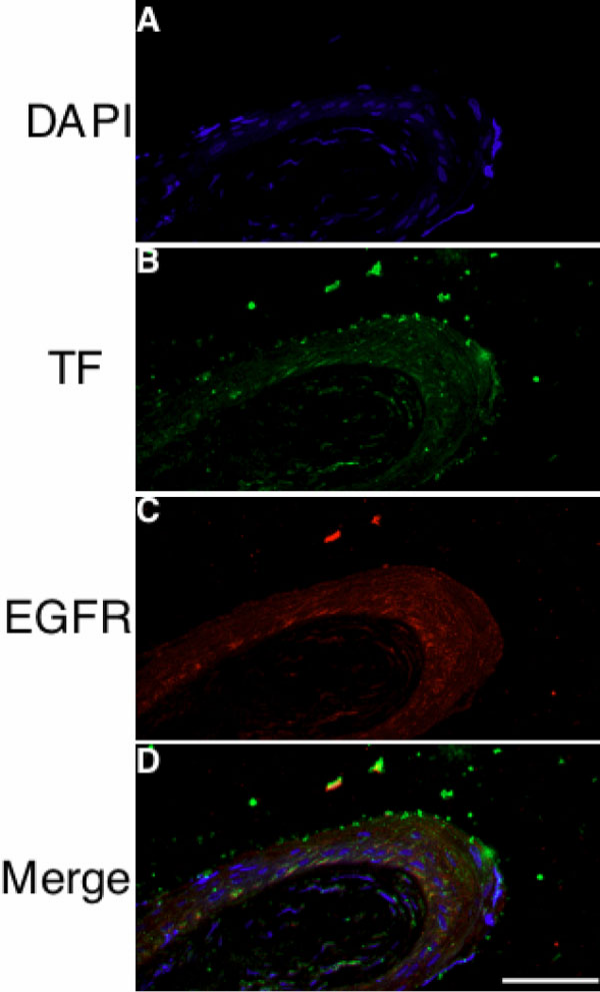
Double staining immunohistochemistry was performed for TF (green) and EGFR (red) in pterygial tissue. Nuclear staining and TF immunoreactivity are shown in **A** and **B**, respectively. **C**, **D**: EGFR immunoreactivity was observed broadly in pterygial epithelial cells. The scale bar represents 50 μm.

Double staining immunohistochemistry for TF and EGFR was also performed in pterygial tissue. EGFR immunoreactivity was observed in pterygial epithelial cells, which was colocalized with TF in preferentially basal cells (Figure 4).

## Discussion

Pterygium has common biologic features with epithelial tumor, as is proliferative tissue and presence of EMT cells [8]. It has been demonstrated that TF functions in tumor initiation, tumor growth, angiogenesis, and metastasis [9-11]. Therefore, we supposed that TF might play a key role in the pathogenesis of pterygium; however, TF expression has yet to be determined in human pterygium. In this study, we demonstrated that TF protein was expressed in pterygial tissues using immunohistochemistry and western blot. Moreover, TF was mainly immunolocalized in pterygial epithelial cells. As shown in Table 1, the number of TF-positive cells was more than half of that of pterygial epithelial cells. In contrast, TF was not expressed in normal conjunctival epithelium. Microvascular endothelial cells showed a weak immunoreaction for TF in both the normal conjunctiva and pterygium, which was not significant. The result showing a significantly higher expression of TF in pterygial epithelium than the normal conjunctiva suggests that TF plays a role in the pathogenesis and development of a pterygium.

EMT is a major factor in pterygium progression [8]. In this study, protein expression of α-SMA, a classic sign of EMT, was observed in several pterygial epithelial cells, where TF immunoreactivity was colocalized on double staining immunohistochemistry. These results indicate that epithelial cells changing to the mesenchymal phenotype expressed TF. In tumor cells, Milsom et al. [9] demonstrated that E-cadherin modulated TF expression, and this could be alleviated by EMT-like changes. These results suggest that TF expression might be controlled by EMT in pterygium as well.

On the other hand, we found that pterygial epithelial cells, showing a negative results for α-SMA, also expressed TF. This suggests that the expression of TF is regulated not only by E-cadherin and EMT, but also by other TF-related molecules such as epidermal growth factor-receptor (EGFR). We and other colleagues previously demonstrated that E-cadherin and EGFR immunoreactivity were shown by pterygial epithelial cells [4,8,13], and we immunohistochemically showed colocalization with TF and EGFR. In human squamous cell carcinoma, the activation of EGFR stimulates TF expression, which is modulated by E-cadherin in vitro, and an E-cadherin-neutralizing antibody led to the upregulation of TF expression [9]. Indeed, this induction of TF was completely inhibited by an EGFR inhibitor [9]. These findings suggest that EGFR signaling pathway may also play an important role in the regulation of TF expression.

It has been demonstrated that subsequent EMT and the activation of TF signaling can induce angiogenesis, tumor growth, and invasion [9]. In fact, invasion to the cornea and angiogenesis are characteristics in the pathobiology of a pterygium. Further investigations of the TF signaling pathway in the pterygium are necessary to clarify TF-mediated pterygial progression. Since targeting TF has been considered to be of therapeutic significance in tumor initiation [9], TF may be a therapeutic molecular target to treat pterygia.
